# Dietary patterns derived by reduced rank regression are associated with lipid disorders among Korean adults: a cross-sectional analysis

**DOI:** 10.1186/s12944-024-02007-1

**Published:** 2024-01-23

**Authors:** Hyun Ah Kim, Hye Ran Shin, SuJin Song

**Affiliations:** Department of Food and Nutrition, Hannam University, 1646 Yuseong-daero, Yuseong-gu, Daejeon, South Korea 34054 Republic of Korea

**Keywords:** Reduced rank regression, Dietary pattern, Dietary fatty acids, Lipid disorders, Korean adults

## Abstract

**Background:**

Lipid disorders are a potent risk factor for cardiovascular diseases. Moreover, the intake of dietary fatty acids has been closely related to blood lipid levels. Therefore, this cross-sectional study examined the associations between dietary patterns related to fatty acid intake and lipid disorders in Korean adults.

**Methods:**

From the 2013–2019 Korea National Health and Nutrition Examination Surveys data, 8399 men and 11404 women (aged ≥ 19 years) were selected. Reduced rank regression was employed to identify dietary patterns from 26 food groups, aiming to explain the maximum variation in the intake of saturated fatty acids (SFA), polyunsaturated fatty acids (PUFA), omega-3 fatty acids, and the PUFA/SFA ratio. Associations of quintiles (Q) of dietary pattern scores with lipid disorders were examined using multiple logistic regression stratified by sex.

**Results:**

Three dietary patterns were identified: dietary pattern 1 showed positive factor loadings for vegetable oils, seasonings, legumes, nuts, and fish; dietary pattern 2 was high in consumption of red meat, bread and snacks, and milk and dairy products; and dietary pattern 3 was rich in fish and milk and dairy products. In men, dietary pattern 3 was inversely associated with elevated triglycerides (Q5 vs. Q1: odds ratio [OR] = 0.82, 95% confidence interval [CI] = 0.69–0.97, *P-*trend = 0.008). In women, dietary pattern 2 was positively associated with elevated total cholesterol (OR = 1.31, 95% CI = 1.12–1.52, *P-*trend < 0.001) but inversely associated with low HDL-cholesterol (OR = 0.70, 95% CI = 0.59–0.83, *P-*trend < 0.001).

**Conclusion:**

In this study, dietary patterns explaining the intake of various types of fatty acids were differentially associated with lipid disorders in Korean adults. Dietary pattern characterized by higher intakes of red meat, bread and snacks and milk and dairy products were positively associated with elevated total cholesterol, whereas dietary pattern rich in fish consumption showed an inverse association with elevated triglycerides. These findings could be instrumental in developing dietary guidelines and strategies for preventing and managing lipid disorders in this population.

## Background

Lipid disorders are characterized by abnormal lipid levels in the blood owing to an increase in total cholesterol, low-density lipoprotein (LDL) cholesterol, or triglycerides, or a decrease in high-density lipoprotein (HDL) cholesterol [1]. Notably, lipid disorders are a potent risk factor for cardiovascular diseases, including atherosclerosis or myocardial infarction, and several studies have reported that they are closely associated with the incidence and mortality of coronary artery disease [2, 3]. Therefore, improving blood lipid profiles is important for the prevention and management of cardiovascular diseases.

Diet is one of the major modifiable risk factors for lipid disorders and cardiovascular diseases [4]. In particular, intake of dietary fatty acids has been reported to be closely related to blood lipid levels [5]. Saturated fatty acids (SFA) have shown the most significant impact on levels of total cholesterol and LDL-cholesterol in the blood. A meta-analysis reported that an increase of 1% in the percentage of energy from SFA intake raised the level of LDL-cholesterol by 0.8–1.6 mg/dL [6]. Similarly, total cholesterol and LDL-cholesterol levels decreased when SFA were replaced by monounsaturated fatty acids (MUFA) or polyunsaturated fatty acids (PUFA) [7, 8]. In contrast, a pooled analysis of results from 49 randomized controlled trials showed that the intake of PUFA was inversely associated with total cholesterol and triglycerides levels, as well as the risks of coronary artery disease and cardiovascular disease [9]. Among PUFA, omega-3 fatty acids, in particular, have a positive impact on the prevention of cardiovascular diseases. Previous studies have shown that the intake of eicosapentaenoic acid (EPA) and docosahexaenoic acid (DHA) reduces serum triglycerides [10, 11]. The ratio of PUFA to SFA is also related to blood lipid levels, and a randomized cross-over study demonstrated that it was inversely associated with the level of LDL-cholesterol [12]. The relationship between dietary fatty acids and lipid disorders has been mainly reported in Western populations, but few studies have investigated the association among Korean adults.

According to the 2021 Korea Health Statistics, the average percentage of energy intake from total fats in Korean adults increased in the last decade from 19.8% to 24.3% [13]. An increase in total fat intake is primarily attributed to heightened intakes of SFA and MUFA in the diet [14, 15]. In addition, the proportion of animal-derived foods as a source of fatty acids in Korean adults has increased, whereas that of plant-based foods and fish has decreased [16]. The prevalence of hypercholesterolemia (≥ 240 mg/dL or receiving lipid-lowering agent) and hyper-LDL-cholesterolemia (≥ 160 mg/dL or receiving lipid-lowering agent) in Korean adults has rapidly increased from 8.8% and 8.7% in 2007 to 19.9% and 19.1% in 2020, respectively, accompanied by changes in dietary behaviors [17]. As of 2020, the prevalence of dyslipidemia (at least one diagnosis of hyper-LDL-cholesterolemia, hypertriglyceridemia, and/or hypo-HDL-cholesterolemia) was 37.7% in Korean adults [17]. Based on the statistics on the cause of death in 2021, the second and fourth leading causes of death among Koreans were heart diseases and cerebrovascular diseases, respectively [18]. Accordingly, effective dietary guidelines and nutritional interventions are required to prevent and manage lipid disorders in Korean adults.

The well-known benefit of analyzing dietary patterns in nutritional epidemiologic studies, as opposed to focusing on individual nutrients or foods, is clear [19]. Compared to factor or cluster analyses, reduced rank regression (RRR) can better predict disease-related dietary patterns by using intermediate markers related to disease and thus is useful in providing nutrition interventions for disease prevention or management [20]. Previous studies used the RRR method to identify dietary patterns and investigate their associations with metabolic syndrome, hypercholesterolemia, and cardiovascular diseases [20–22]. However, few studies have investigated the associations of RRR-derived dietary patterns with lipid disorders.

Because the relationship between dietary fatty acids and lipid disorders varies depending on the type and amount of dietary fatty acids, the RRR method can be used to derive dietary patterns that explain the intake of various types of fatty acids and to investigate the association between dietary patterns and lipid disorders. Using the RRR method might be helpful to obtain information on overall food components for the prevention and management of lipid disorders and present effective dietary strategies to improve lipid levels. This study hypothesized that dietary patterns explaining the intake of various types of fatty acids are differently associated with lipid disorders in Korean adults. Therefore, this study used the RRR method to derive dietary patterns related to the intake of dietary fatty acids in healthy Korean adults and investigated the relationship between dietary patterns and lipid disorders.

## Methods

### Study data and participants

This study utilized the 2013–2019 Korea National Health and Nutrition Examination Survey (KNHANES) data. The KNHANES is the national statistics collected annually by the Korea Disease Control and Prevention Agency (KDCA) to assess and monitor the health and nutritional status of Koreans. A nationally representative sample of the Korean population, aged one year or older, is derived using a stratified clustered sampling design. Within this sample, individuals undergo a health interview survey, a health examination survey, and a nutrition survey [23].

In this study, we selected data from 44,029 adults aged 19 years and older who participated in the KNHANES from 2013 to 2019. We excluded adults meeting any of the following criteria: 1) absence in 24-h dietary recalls (5,334 participants), 2) daily energy intake below 500 kcal or above 5,000 kcal [24–26] (708 participants), 3) fasting duration less than 8 h before the blood test (3,544 participants), 4) missing anthropometry and biochemistry data (1,556 participants), 5) incomplete sociodemographic or lifestyle information (1,968 participants), and 6) pregnancy or breastfeeding status. Furthermore, adults with a history of hypertension, diabetes, or dyslipidemia, or those on related medications (9,660 participants), were also excluded. Finally, 19,803 individuals (8,399 men and 11,404 women) were included in the data analysis. The KNHANES was conducted in accordance with the guidelines of the Declaration of Helsinki. The KNHANES conducted in 2013–2014 and 2018–2019 were reviewed and approved by the institutional review board (IRB) of the KDCA (IRB No. 2013-07CON-03-4C, 2013-12EXP-03-5C, 2018–01-03-P-A, and 2018–01-03-C-A). The KNHANES conducted in 2015–2017 qualified for an exemption issued by the KDCA IRB. All study participants provided written informed consent.

### Assessment of dietary patterns

This study utilized food intake data gathered via 24-h dietary recalls from one day during the KNHANES nutrition survey. These recalls were conducted 7 to 12 days subsequent to the health interview and examination surveys. During the process of dietary assessment, a standardized and structured interview was conducted for each participant by trained investigators. The investigators visited households to interview participants directly about the types and quantities of all foods, drinks, and dietary supplements consumed in the 24 h preceding the interview. Food models, pictures, and other visual aids were used to help participants recall quantities of foods and beverages consumed [27].

Energy and nutrient intakes were calculated using the national standard food composition table provided by the Korea Rural Development Administration [28, 29]. To calculate dietary fatty acid intake, a fatty acid database containing the fatty acid content of Korean foods was used [30]. The intakes of macronutrients and each type of fatty acid (SFA, MUFA, PUFA, omega-3, and omega-6 fatty acids) were presented by calculating the percentage of energy intake (% E) from each nutrient, and the ratio of PUFA to SFA intake was also computed.

To derive dietary patterns in this study, the RRR method was used. The intake of food groups was used as predictor variables to extract dietary patterns. Foods that the participants consumed were classified into 26 groups (Table 1), and the percentage of energy intake from each food group was calculated. As the RRR determines combinations of foods (dietary patterns) that explain the maximum variation of response variables [31], four fatty acid intake variables associated with lipid disorders were selected as response variables based on the results of previous studies: 1) SFA (% E) [7, 8], 2) PUFA (% E) [9], 3) omega-3 fatty acids (% E) [32–34], and 4) the PUFA to SFA intake ratio [8]. The number of dietary patterns obtained via the RRR method is identical to the number of response variables [31]. Among the four dietary patterns extracted by the analysis, three dietary patterns were finally selected based on the explained variation and interpretation of each dietary pattern. Four dietary patterns extracted in this study explained 32.2%, 25.0%, 2.2%, and 0.2% of variation in the response variables, respectively. The fourth dietary pattern was not examined further as the pattern did not significantly add to explaining the variation in the specified response variables. The pattern score of each dietary pattern obtained during dietary pattern extraction was categorized into quintiles (Q) by sex for further analysis. In each dietary pattern, Q1 represents the lowest value, while Q5 represents the highest value.

**Table 1:** Description of food groups included in the dietary pattern analyses

Food group	Main food items
White rice	White rice, rice cakes, porridge
Other grains	Oat, barley, sorghum, millet, brown rice, corn
Noodles and dumplings	Noodles, instant noodles (*Ramyeon*), wheat flour, dumplings
Bread and snacks	Bread, pizza, hamburgers, cakes, sandwiches, pies, pastries, croquettes, snacks
Potatoes and starches	Potatoes, sweet potatoes
Sugar and sweets	Honey, candy, jam, sugar, jelly, chocolate, chewing gum, syrup, caramel
Legumes	Legumes, beans, tofu, soy milk
Nuts	Nuts, seeds
Vegetables	Vegetables
Kimchi and pickles	Kimchi (traditional fermented cabbage), pickles
Mushrooms	Mushrooms
Fruits	Fresh fruit, dried fruit, canned fruit
Red meat	Pork, beef
White meat	Chicken, duck, turkey
Processed meat	Ham, sausage, bacon
Eggs	Eggs
Fish	Fish, canned fish, dried fish, salted fish
Shellfish	Shellfish, squid, octopus
Seaweed	Seaweed
Milk and dairy products	Milk, ice cream, cheese, cream, yogurt
Vegetable oil	Soybean oil, sesame oil, corn oil, olive oil, grape seed oil, avocado oil
Animal oil	Lard, butter, coffee creamer
Alcohol	Wine, beer, rice wine (*Makgeolli*), whiskey
Sugar-sweetened beverages	Soft drinks, artificially sweetened soft drinks, fruit juice
Seasonings	Seasonings, dressing, mayonnaise, sauce
Coffee	Coffee, coffee mix, coffee beverages

### Diagnosis of lipid disorders

Blood test data obtained from the health examination of KNHANES were used to diagnose lipid disorders. Blood samples were collected from the participants after they had fasted for at least 8 h, and lipid indices, including total cholesterol, LDL-cholesterol, HDL-cholesterol, and triglycerides, were analyzed. The enzymatic method was used to measure total cholesterol and triglycerides levels, and the homogeneous enzymatic colorimetric method was used to measure LDL-cholesterol and HDL-cholesterol levels. All blood samples were analyzed using Hitachi Automatic Analyzer 7600–210 (Hitachi, Tokyo, Japan) [35].

In this study, lipid disorders included elevated total cholesterol, elevated LDL-cholesterol, low HDL-cholesterol, and elevated triglycerides. Because this study included healthy adults as study subjects, each lipid disorder was defined as the borderline criteria specified in the classification of dyslipidemia provided by the Korean Society of Lipid and Atherosclerosis [36]. Accordingly, the ranges for lipid disorders were as follows: elevated total cholesterol ≥ 200 mg/dL; elevated LDL-cholesterol ≥ 130 mg/dL; low HDL-cholesterol < 40 mg/dL in men and < 50 mg/dL in women; and elevated triglycerides ≥ 150 mg/dL [36].

### Measurement of sociodemographic and lifestyle variables

Information on sociodemographic and lifestyle variables of the participants was collected via the health interview survey. Household income was classified as low, medium–low, medium–high, and high, and education was classified as ≤ elementary school, middle school, high school, and ≥ college. Smoking status was classified as “yes” if the participants were currently smoking and “no” if they had smoked in the past or never smoked. Drinking status was classified as “yes” if the participants had consumed at least one glass of alcohol per month for the past year and “no” if it was less than one glass or they had never consumed alcohol. Physical activity was classified as “yes” for participants who had engaged in physical activity on at least three days in the previous week, and as “no” for those who had done so on fewer than three days. Body mass index (BMI; kg/m^2^) was calculated using height and weight measurements taken by the trained investigators.

### Statistical analyses

All data analyses of this study were performed using SAS 9.4 (Statistical Analysis System version 9.4, SAS Institute, Cary, NC, USA). The RRR method was employed using the partial least squares (PLS) procedure in SAS to derive the dietary patterns. Given the complex sampling design of the KNHANES, the weight, stratification, and cluster variables were considered in all analyses. For the participant characteristics, continuous variables were presented as the mean and standard error (mean ± SE), and categorical variables were presented as percentages (%). Differences in variables by sex were analyzed using a t-test or chi-square test. According to the quintiles of the dietary pattern scores, categorical variables such as sociodemographic and lifestyle variables were presented as percentages (%), and distribution differences were compared using the Rao-Scott chi-square test. Continuous variables, such as age, BMI, and nutrient intake, were presented as the mean ± SE, and a general linear model was used to compare differences in means. Multiple logistic regression analysis was used to determine the relationships between dietary patterns and lipid disorders stratified by sex in response to prior research that have observed significant disparities in lipid disorders, such as dyslipidemia and cardiovascular diseases, between men and women [37, 38]. Odds ratios (ORs) and 95% confidence intervals (CIs) were calculated for each type of lipid disorder according to the quintiles of dietary pattern score. For all analysis models, age, residence, household income, education level, smoking status, drinking status, physical activity, energy intake, BMI, and menopausal status were included as adjustment variables. *P-*trends were calculated via multiple logistic regression analysis with the medians of the quintiles of dietary pattern scores as continuous variables. A *P-*value < 0.05 was considered statistically significant.

## Results

### Characteristics of the study participants

The participants’ characteristics are presented by sex in Table 2. Compared to men, women had a lower BMI, lived predominantly in urban areas, and had a lower education level. Furthermore, the proportion of women who were currently smoking, drinking alcohol, or performing physical activity was lower than that of men. Although women had a lower total energy intake than men did, their percentage of energy intake from carbohydrates was higher, while that of energy intake from protein was lower than those of men. There was no difference in the total fat intake between men and women, but men showed a higher intake of MUFA and omega-3 fatty acids than did women. In contrast, the intake of SFA, PUFA, and omega-6 fatty acids was not significantly different between men and women. The prevalence of all types of lipid disorders was higher in men than in women, but that of low HDL-cholesterol was higher in women than in men.

**Table 2:** Study participants’ characteristics by sex

**Variables**	**Total (*n* = 19803)**	**Men (*n* = 8399)**	**Women (*n* = 11404)**	*P-*value^1^
Age (years)	41.5 ± 0.1	41.5 ± 0.2	41.4 ± 0.2	0.747
BMI (kg/m^2^)	23.4 ± 0.03	24.3 ± 0.04	22.7 ± 0.04	< 0.001
Residence				
Urban	86.2	85.3	87.0	0.001
Rural	13.8	14.7	13.0	
Household income				
Low	10.0	9.9	10.1	0.267
Medium–low	24.0	23.6	24.4	
Medium–high	31.6	32.3	31.0	
High	34.4	34.2	34.5	
Education				
≤ Elementary school	7.0	6.2	7.8	< 0.001
Middle school	6.3	6.1	6.5	
High school	39.2	40.2	38.3	
≥ College	47.5	47.6	47.4	
Current smoking	21.4	37.8	5.9	< 0.001
Current alcohol consumption	61.8	73.1	51.1	< 0.001
Physical activity	16.9	23.4	10.7	< 0.001
Menopausal status				
No			76.3	
Yes			23.7	
Total energy (kcal)	2071 ± 8.0	2386 ± 11.6	1753 ± 7.6	< 0.001
Carbohydrate (% E)	62.8 ± 0.1	62.2 ± 0.2	63.3 ± 0.1	< 0.001
Protein (% E)	15.3 ± 0.1	15.7 ± 0.1	14.8 ± 0.1	< 0.001
Fat (% E)	22.0 ± 0.1	22.1 ± 0.1	21.9 ± 0.1	0.228
SFA (% E)	6.9 ± 0.03	6.9 ± 0.05	6.9 ± 0.04	0.999
MUFA (% E)	7.0 ± 0.04	7.1 ± 0.1	6.9 ± 0.04	0.017
PUFA (% E)	5.5 ± 0.03	5.5 ± 0.04	5.5 ± 0.03	0.831
Omega-3 FA (% E)	0.8 ± 0.01	0.8 ± 0.01	0.8 ± 0.01	0.005
Omega-6 FA (% E)	4.7 ± 0.02	4.7 ± 0.03	4.7 ± 0.03	0.243
PUFA:SFA	1.0 ± 0.01	1.0 ± 0.01	1.0 ± 0.01	0.019
Elevated total cholesterol^2^	38.7	40.2	37.3	< 0.001
Elevated LDL-cholesterol^3^	8.4	11.3	5.8	< 0.001
Low HDL-cholesterol^4^	26.8	22.2	31.1	< 0.001
Elevated triglycerides^5^	24.8	35.8	14.3	< 0.001

Values are presented as the mean ± standard error or %

All analyses accounted for the complex sampling design and appropriate sampling weights

*Abbreviations*: *BMI *body mass index., *% E* percentage of total energy, *SFA *saturated fatty acids, *MUFA *monounsaturated fatty acids, *PUFA *polyunsaturated fatty acids, *FA *fatty acids, *LDL *low-density lipoprotein, *HDL *high-density lipoprotein

^1^ *P-*values were obtained from the independent t-test or Rao-Scott chi-square test

^2^ Elevated total cholesterol (≥ 200 mg/dL)

^3^ Elevated LDL-cholesterol (≥ 130 mg/dL)

^4^ Low HDL-cholesterol (men < 40 mg/dL, women < 50 mg/dL)

^5^ Elevated triglycerides (≥ 150 mg/dL)

### Characteristics of extracted dietary patterns

**Fig. 1:**
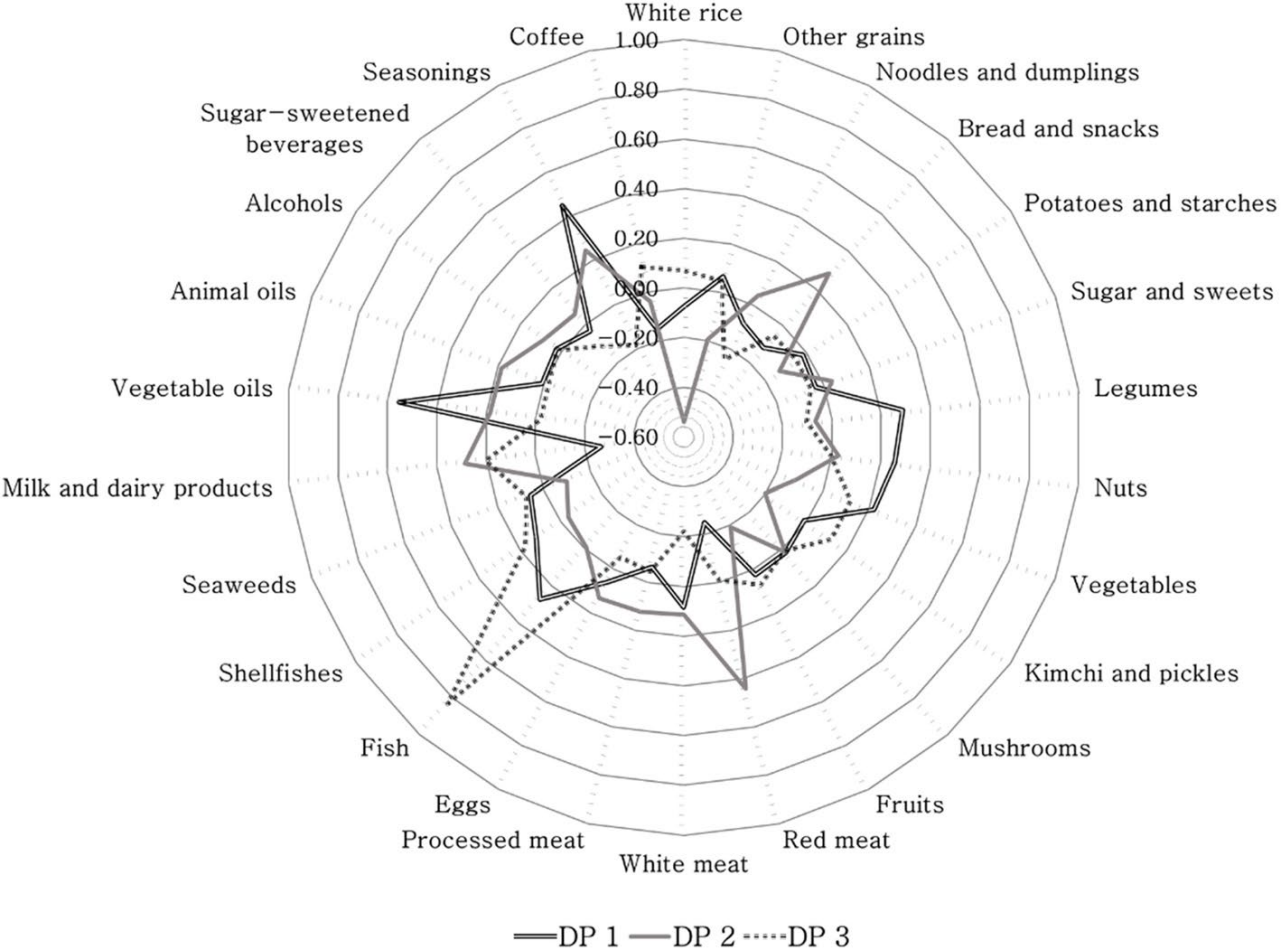
Factor loadings matrix for the three dietary patterns related to fatty acid intake extracted via reduced rank regression Abbreviations: *DP* dietary pattern

In Korean adults, three dietary patterns related to fatty acid intake were identified via the RRR method. The percentages of variation explained by each dietary pattern and the correlation coefficients for the response variables in each dietary pattern are presented in Table 3. Dietary pattern 1 was positively correlated with PUFA (*r* = 0.60) and omega-3 fatty acids (*r* = 0.46), but negatively correlated with SFA (*r* = -0.24). Dietary pattern 2 was positively correlated with SFA (*r* = 0.80) and PUFA (*r* = 0.50) whereas negatively associated with the PUFA to SFA ratio (*r* = -0.29). Dietary pattern 3 was positively correlated with omega-3 fatty acids (*r* = 0.87), but inversely associated with PUFA (*r* = -0.42). The factor loading matrix of these patterns is provided in Fig. 1. In dietary pattern 1, consumption of vegetable oil, seasonings, legumes, fish, and nuts was high, whereas that of milk and dairy products and red meat was low. In dietary pattern 2, consumption of red meat, milk and dairy products, and bread and snacks was high, whereas negative factor loadings were observed for white rice and kimchi and pickles. In dietary pattern 3, higher factor loadings of fish and milk and dairy products but negative factor loadings for noodles and dumplings and white meat were observed.

**Table 3:** Explained variation (%) in food intakes and response variables for each dietary pattern and correlation coefficient between dietary patterns and response variables

Dietary patterns	Explained variation (%)						Correlation coefficient			
	Total		Response variables							
	Food intakes	Response variables	SFA (% E)	PUFA (% E)	Omega-3 FA (% E)	PUFA:SFA ratio	SFA (% E)	PUFA (% E)	Omega-3 FA (% E)	PUFA:SFA ratio
Dieatary pattern 1	4.84	32.2	7.7	46.6	27.4	47.2	-0.24	0.60	0.46	0.61
Dieatary pattern 2	5.63	25.0	71.7	71.8	29.8	55.8	0.80	0.50	0.15	-0.29
Dieatary pattern 3	4.04	2.2	71.7	73.3	36.5	56.3	0.01	-0.42	0.87	-0.24

*Abbreviations*: *% E* percentage of total energy, *SFA* saturated fatty acids, *PUFA* polyunsaturated fatty acids, *FA* fatty acids

### Participants’ characteristics by dietary patterns score

The participants’ characteristics by dietary pattern score are shown in Table 4. In both men and women, participants in the highest group of dietary pattern 1 score, compared to those in the lowest group, were older, lived in urban areas, and had higher education and income levels but were less likely to be current smokers. With an increase in the score of dietary pattern 2, the ages of participants became lower, while the proportion of participants living in urban areas increased along with education and income levels. In addition, the percentages of those who smoked, drank, and performed physical activity increased. The BMI in men increased as the score of dietary pattern 2 increased, whereas that of women decreased. Compared to individuals with the lowest score of dietary pattern 3, those with the highest score of dietary pattern 3 in both men and women were older and had lower education levels. Among men, the proportion of those living in urban areas decreased as the score of dietary pattern 3 increased.

**Table 4:** Study participants’ characteristics across quintiles (Q) of dietary pattern scores by sex

**Characteristics**	**Men (*n* = 8399)**				**Women (*n* = 11404)**			
	Q1 (*n* = 1679)	Q3 (*n* = 1680)	Q5 (*n* = 1680)	*P*-value^1^	Q1 (*n* = 2280)	Q3 (*n* = 2281)	Q5 (*n* = 2281)	*P*-value^1^
**Dietary pattern 1**								
Age (years)	38.6 ± 0.4	41.9 ± 0.4	41.3 ± 0.4	< 0.001	38.3 ± 0.3	42.7 ± 0.3	41.8 ± 0.3	< 0.001
BMI (kg/m^2^)	24.3 ± 0.1	24.2 ± 0.1	24.6 ± 0.1	0.012	22.6 ± 0.1	22.6 ± 0.1	22.5 ± 0.1	0.869
Residence								
Urban	85.4	85.9	89.0	0.005	88.8	87.1	90.3	0.006
Rural	14.6	14.1	11.0		11.2	12.9	9.7	
Household income								
Low	10.6	9.7	7.9	< 0.001	10.5	10.6	8.0	< 0.001
Medium–low	25.6	23.3	19.2		25.5	22.9	21.7	
Medium–high	31.1	34.2	31.4		30.9	32.2	31.6	
High	32.6	32.7	41.5		33.1	34.3	38.8	
Education								
≤ Elementary school	5.6	6.9	4.7	< 0.001	6.6	9.4	6.1	0.001
Middle school	5.2	5.5	5.9		6.0	6.6	6.2	
High school	44.8	40.1	34.9		40.4	38.0	36.2	
≥ College	44.4	47.5	54.5		47.1	45.9	51.4	
Current smoking	44.4	37.9	32.4	< 0.001	7.4	5.3	5.1	0.051
Current alcohol consumption	69.9	73.6	74.8	0.009	53.5	48.9	52.9	0.048
Physical activity	21.4	22.7	24.0	0.226	10.6	9.3	11.6	0.034
Menopausal status								
No					82.9	73.1	75.2	< 0.001
Yes					17.1	26.9	24.8	
**Dietary pattern 2**								
Age (years)	49.9 ± 0.5	41.6 ± 0.4	34.5 ± 0.3	< 0.001	50.0 ± 0.4	40.7 ± 0.3	35.6 ± 0.3	< 0.001
BMI (kg/m^2^)	24.0 ± 0.1	24.2 ± 0.1	24.7 ± 0.1	< 0.001	23.0 ± 0.1	22.6 ± 0.1	22.4 ± 0.1	< 0.001
Residence								
Urban	79.9	86.6	90.2	< 0.001	83.4	89.0	90.9	< 0.001
Rural	20.1	13.4	9.8		16.6	11.0	9.1	
Household income								
Low	17.1	8.7	7.4	< 0.001	17.2	7.8	7.1	< 0.001
Medium–low	25.6	21.6	20.6		26.3	22.9	21.5	
Medium–high	28.3	34.3	31.1		27.8	33.8	31.9	
High	29.0	35.5	40.8		28.8	35.5	39.5	
Education								
≤ Elementary school	16.1	4.5	1.3	< 0.001	21.5	4.9	2.2	< 0.001
Middle school	11.5	6.2	2.2		10.5	6.2	3.1	
High school	35.7	39.7	42.9		38.1	39.4	37.1	
≥ College	36.6	49.6	53.6		29.8	49.4	57.5	
Current smoking	30.2	39.9	39.0	< 0.001	3.4	5.9	7.5	< 0.001
Current alcohol consumption	63.9	75.6	76.6	< 0.001	38.3	54.2	59.9	< 0.001
Physical activity	21.8	21.0	25.6	0.028	9.4	9.5	12.6	0.002
Menopausal status								
No					54.0	79.0	88.2	< 0.001
Yes					46.0	21.0	11.8	
**Dietary pattern 3**								
Age (years)	36.6 ± 0.4	42.0 ± 0.4	44.6 ± 0.4	< 0.001	38.0 ± 0.3	42.0 ± 0.3	42.9 ± 0.3	< 0.001
BMI (kg/m^2^)	24.4 ± 0.1	24.1 ± 0.1	24.4 ± 0.1	0.501	22.4 ± 0.1	22.8 ± 0.1	22.6 ± 0.1	0.238
Residence								
Urban	88.8	83.8	84.6	< 0.001	89.2	87.8	87.4	0.436
Rural	11.2	16.2	15.4		10.8	12.2	12.6	
Household income								
Low	10.5	8.9	9.0	0.099	10.4	9.9	9.7	0.296
Medium–low	24.5	24.5	21.7		24.9	22.5	23.9	
Medium–high	31.3	31.9	31.5		30.3	32.1	28.9	
High	33.6	34.7	37.7		34.4	35.5	37.5	
Education								
≤ Elementary school	4.2	5.6	6.7	< 0.001	6.8	7.6	7.1	0.005
Middle school	4.0	6.7	7.3		5.1	7.3	7.9	
High school	44.4	41.5	35.5		38.3	39.5	37.5	
≥ College	47.3	46.2	50.6		49.9	45.6	47.5	
Current smoking	35.7	39.7	38.7	0.145	6.7	5.0	6.0	0.342
Current alcohol consumption	72.9	74.2	74.4	0.299	53.8	50.2	51.5	0.255
Physical activity	25.9	23.8	22.4	0.068	10.6	10.3	11.4	0.615
Menopausal status								
No					81.1	74.8	73.2	< 0.001
Yes					18.9	25.2	26.8	

Values are presented as the mean ± standard error or %

All analyses accounted for the complex sampling design effect and appropriate sampling weights

*Abbreviations*: *BMI *body mass index

^1^ *P-*values were obtained from the Rao-Scott chi-square test

### Participants’ nutrient intake by dietary pattern score

Table 5 shows the energy and nutrient intake of the participants by dietary pattern score. In both men and women, individuals with the highest score of dietary pattern 1 showed higher intakes of energy, protein, PUFA, omega-3 fatty acids, and omega-6 fatty acids but lower intakes of carbohydrates, SFA, and MUFA than did those with the lowest score. As the score of dietary pattern 2 increased, energy, protein, total fats, and all fatty acids increased in both men and women, but the intake of carbohydrates and the ratio of PUFA to SFA intake decreased. In dietary pattern 3, as the score increased in both men and women, the intakes of energy, PUFA, omega-6 fatty acids, and the ratio of PUFA to SFA intake decreased, whereas the consumption of carbohydrates, protein, and omega-3 fatty acids increased. However, as the score of dietary pattern 3 increased, the intake of SFA significantly decreased in men and increased in women.

**Table 5:** Study participants’ energy and nutrient intake across quintiles (Q) of dietary pattern scores by sex

**Energy and nutrient intake**	**Men (*n* = 8399)**				**Women (*n* = 11404)**			
	Q1 (*n* = 1679)	Q3 (*n* = 1680)	Q5 (*n* = 1680)	*P-*value^1^	Q1 (*n* = 2280)	Q3 (*n* = 2281)	Q5 (*n* = 2281)	*P-*value^1^
**Dietary pattern 1**								
Total energy (kcal)	2330 ± 25.1	2386 ± 23.5	2449 ± 26.4	0.001	1679 ± 15.9	1723 ± 15.1	1838 ± 17.5	< 0.001
Carbohydrates (% E)	60.7 ± 0.4	64.8 ± 0.3	58.1 ± 0.3	< 0.001	61.9 ± 0.3	66.0 ± 0.3	58.0 ± 0.2	< 0.001
Protein (% E)	14.6 ± 0.1	15.5 ± 0.1	17.5 ± 0.2	< 0.001	13.9 ± 0.1	14.6 ± 0.1	16.7 ± 0.1	< 0.001
Fat (% E)	24.7 ± 0.3	19.7 ± 0.2	24.5 ± 0.2	0.263	24.2 ± 0.3	19.4 ± 0.2	25.3 ± 0.2	< 0.001
SFA (% E)	9.4 ± 0.1	6.1 ± 0.1	5.9 ± 0.1	< 0.001	9.5 ± 0.1	6.1 ± 0.1	6.0 ± 0.1	< 0.001
MUFA (% E)	8.6 ± 0.1	6.3 ± 0.1	7.3 ± 0.1	< 0.001	8.0 ± 0.1	6.2 ± 0.1	7.6 ± 0.1	0.029
PUFA (% E)	3.8 ± 0.1	4.9 ± 0.05	8.8 ± 0.1	< 0.001	3.7 ± 0.05	4.8 ± 0.05	9.1 ± 0.1	< 0.001
Omega-3 FA (% E)	0.4 ± 0.01	0.7 ± 0.01	1.4 ± 0.02	< 0.001	0.5 ± 0.04	0.7 ± 0.01	1.5 ± 0.02	< 0.001
Omega-6 FA (% E)	3.4 ± 0.05	4.2 ± 0.05	7.4 ± 0.1	< 0.001	3.3 ± 0.04	4.1 ± 0.04	7.6 ± 0.1	< 0.001
PUFA:SFA	0.4 ± 0.01	0.9 ± 0.01	1.6 ± 0.02	< 0.001	0.4 ± 0.01	1.0 ± 0.01	1.7 ± 0.02	< 0.001
**Dietary pattern 2**								
Total energy (kcal)	1958 ± 22.2	2421 ± 22.6	2706 ± 27.9	< 0.001	1550 ± 15.5	1778 ± 15.1	1953 ± 19.1	< 0.001
Carbohydrates (% E)	76.0 ± 0.2	63.6 ± 0.2	49.3 ± 0.3	< 0.001	77.2 ± 0.1	64.3 ± 0.1	50.2 ± 0.2	< 0.001
Protein (% E)	12.9 ± 0.1	15.9 ± 0.2	17.3 ± 0.1	< 0.001	12.1 ± 0.1	14.9 ± 0.1	16.5 ± 0.1	< 0.001
Fat (% E)	11.1 ± 0.1	20.5 ± 0.1	33.4 ± 0.2	< 0.001	10.7 ± 0.1	20.8 ± 0.1	33.2 ± 0.2	< 0.001
SFA (% E)	3.2 ± 0.05	6.2 ± 0.1	11.0 ± 0.1	< 0.001	3.0 ± 0.04	6.4 ± 0.1	11.2 ± 0.1	< 0.001
MUFA (% E)	3.1 ± 0.05	6.4 ± 0.1	11.4 ± 0.1	< 0.001	3.1 ± 0.04	6.5 ± 0.1	11.0 ± 0.1	< 0.001
PUFA (% E)	3.2 ± 0.04	5.5 ± 0.1	7.3 ± 0.1	< 0.001	3.2 ± 0.04	5.5 ± 0.1	7.3 ± 0.1	< 0.001
Omega-3 FA (% E)	0.6 ± 0.02	0.8 ± 0.02	0.9 ± 0.02	< 0.001	0.6 ± 0.02	0.8 ± 0.02	1.0 ± 0.04	< 0.001
Omega-6 FA (% E)	2.6 ± 0.04	4.6 ± 0.05	6.4 ± 0.1	< 0.001	2.6 ± 0.03	4.6 ± 0.05	6.4 ± 0.1	< 0.001
PUFA:SFA	1.2 ± 0.02	1.0 ± 0.02	0.8 ± 0.01	< 0.001	1.3 ± 0.02	1.0 ± 0.01	0.7 ± 0.01	< 0.001
**Dietary pattern 3**								
Total energy (kcal)	2445 ± 26.1	2402 ± 23.9	2246 ± 23.5	< 0.001	1797 ± 17.4	1774 ± 17.0	1681 ± 16.5	< 0.001
Carbohydrates (% E)	59.8 ± 0.3	63.4 ± 0.3	61.5 ± 0.3	< 0.001	61.2 ± 0.3	65.1 ± 0.3	61.3 ± 0.3	< 0.001
Protein (% E)	16.1 ± 0.2	14.7 ± 0.1	17.7 ± 0.2	< 0.001	15.1 ± 0.1	13.9 ± 0.1	16.8 ± 0.1	< 0.001
Fat (% E)	24.1 ± 0.3	21.9 ± 0.3	20.8 ± 0.3	0.078	23.6 ± 0.2	21.0 ± 0.2	21.9 ± 0.2	0.236
SFA (% E)	6.9 ± 0.1	7.1 ± 0.1	6.5 ± 0.1	< 0.001	6.6 ± 0.1	6.8 ± 0.1	7.2 ± 0.1	< 0.001
MUFA (% E)	7.4 ± 0.1	7.3 ± 0.1	6.4 ± 0.1	0.545	7.2 ± 0.1	6.8 ± 0.1	6.8 ± 0.1	0.026
PUFA (% E)	7.0 ± 0.1	4.9 ± 0.1	5.4 ± 0.1	< 0.001	7.2 ± 0.1	4.9 ± 0.1	5.3 ± 0.1	< 0.001
Omega-3 FA (% E)	0.8 ± 0.01	0.7 ± 0.01	1.2 ± 0.02	< 0.001	0.9 ± 0.02	0.7 ± 0.01	1.2 ± 0.02	< 0.001
Omega-6 FA (% E)	6.2 ± 0.1	4.3 ± 0.1	4.1 ± 0.1	< 0.001	6.4 ± 0.1	4.2 ± 0.1	4.2 ± 0.1	< 0.001
PUFA:SFA	1.2 ± 0.02	0.9 ± 0.02	1.0 ± 0.02	< 0.001	1.3 ± 0.02	0.9 ± 0.01	1.0 ± 0.02	< 0.001

Values are presented as the mean ± standard error

All analyses accounted for the complex sampling design effect and appropriate sampling weights

^1^ *P-*value from the general linear model across quintiles of dietary pattern scores adjusted for age, residence, household income, education, current smoking, current alcohol consumption, physical activity, total energy intake, body mass index, and menopausal status

Abbreviations: *% E* percentage of total energy, *SFA* saturated fatty acids, *MUFA* monounsaturated fatty acids, *PUFA* polyunsaturated fatty acids, *FA* fatty acids

### Association between dietary patterns and lipid disorders

Table 6 shows the association between the three dietary patterns and lipid disorders. After adjusting for age, residence, household income, education, smoking status, drinking status, physical activity, energy intake, BMI, and menopausal status, dietary pattern 1 showed no significant relationships with lipid disorders in either men or women. Compared to women with the lowest score of dietary pattern 2, those with the highest score of dietary pattern 2 showed significantly higher OR for elevated total cholesterol (OR = 1.31, 95% CI = 1.12–1.52, *P* for trend < 0.001) but reduced OR for low HDL-cholesterol (OR = 0.70, 95% CI = 0.59–0.83, *P* for trend < 0.001). Among men, a significant linear trend in decreasing ORs for elevated triglycerides was observed across the quintiles of dietary pattern 2 scores (*P* for trend = 0.003), but the OR value in the highest quintile was not statistically significant (Q5 vs. Q1: OR = 0.87, 95% CI = 0.72–1.06). Compared to the group with the lowest score of dietary pattern 3, the OR of elevated triglycerides was 0.82 in the group with the highest dietary pattern 3 scores in men (95% CI = 0.69–0.97, *P* for trend = 0.008), whereas there was no significant association between dietary pattern 3 and lipid disorders in women.

**Table 6:** Odds ratios for lipid disorders across quintiles (Q) of dietary pattern scores by sex^a^

	Q1	Q2	Q3	Q4	Q5	
	OR (95% CI)	OR (95% CI)	OR (95% CI)	OR (95% CI)	OR (95% CI)	*P* for trend
**Dietary pattern 1**						
**Men**						
Elevated total cholesterol^b^	1.00 (ref.)	0.92 (0.79, 1.08)	1.08 (0.92, 1.27)	1.18 (1.00, 1.40)	1.07 (0.91, 1.25)	0.090
Elevated LDL-cholesterol^c^	1.00 (ref.)	0.98 (0.76, 1.27)	1.08 (0.84, 1.40)	1.13 (0.88, 1.45)	1.06 (0.82, 1.37)	0.475
Low HDL-cholesterol^d^	1.00 (ref.)	1.09 (0.90, 1.32)	1.01 (0.84, 1.23)	0.91 (0.75, 1.11)	1.03 (0.85, 1.24)	0.721
Elevated triglycerides^e^	1.00 (ref.)	1.14 (0.96, 1.36)	1.12 (0.95, 1.32)	1.12 (0.94, 1.33)	1.03 (0.88, 1.22)	0.981
**Women**						
Elevated total cholesterol	1.00 (ref.)	1.03 (0.89, 1.19)	1.00 (0.86, 1.15)	1.09 (0.94, 1.27)	1.01 (0.87, 1.17)	0.781
Elevated LDL-cholesterol	1.00 (ref.)	0.99 (0.75, 1.32)	1.00 (0.74, 1.35)	0.85 (0.62, 1.16)	1.11 (0.84, 1.47)	0.688
Low HDL-cholesterol	1.00 (ref.)	1.10 (0.95, 1.27)	1.08 (0.93, 1.25)	0.97 (0.84, 1.13)	1.01 (0.88, 1.17)	0.601
Elevated triglycerides	1.00 (ref.)	1.26 (1.03, 1.55)^*^	1.08 (0.88, 1.33)	1.05 (0.85, 1.30)	0.95 (0.77, 1.18)	0.188
**Dietary pattern 2**						
**Men**						
Elevated total cholesterol	1.00 (ref.)	1.20 (1.02, 1.42)^*^	1.05 (0.88, 1.24)	1.17 (0.99, 1.38)	1.17 (0.98, 1.40)	0.184
Elevated LDL-cholesterol	1.00 (ref.)	1.06 (0.82, 1.38)	0.89 (0.68, 1.16)	0.90 (0.68, 1.18)	0.92 (0.68, 1.23)	0.348
Low HDL-cholesterol	1.00 (ref.)	0.94 (0.77, 1.14)	0.84 (0.69, 1.03)	0.93 (0.77, 1.14)	0.87 (0.70, 1.08)	0.287
Elevated triglycerides	1.00 (ref.)	1.16 (0.98, 1.38)	1.09 (0.91, 1.30)	0.84 (0.70, 1.00)^*^	0.87 (0.72, 1.06)	0.003
**Women**						
Elevated total cholesterol	1.00 (ref.)	1.15 (1.00, 1.34)	1.25 (1.08, 1.44)^**^	1.42 (1.21, 1.66)^***^	1.31 (1.12, 1.52)^***^	< 0.001
Elevated LDL-cholesterol	1.00 (ref.)	0.83 (0.64, 1.08)	0.77 (0.58, 1.02)	0.87 (0.64, 1.17)	0.76 (0.54, 1.05)	0.150
Low HDL-cholesterol	1.00 (ref.)	0.87 (0.75, 1.02)	0.75 (0.64, 0.88)^***^	0.77 (0.66, 0.90)^**^	0.70 (0.59, 0.83)^***^	< 0.001
Elevated triglycerides	1.00 (ref.)	0.98 (0.82, 1.19)	1.02 (0.84, 1.24)	1.08 (0.88, 1.32)	0.93 (0.74, 1.17)	0.776
**Dietary pattern 3**						
**Men**						
Elevated total cholesterol	1.00 (ref.)	1.00 (0.85, 1.18)	1.19 (1.01, 1.39)^*^	1.06 (0.90, 1.24)	1.06 (0.90, 1.24)	0.520
Elevated LDL-cholesterol	1.00 (ref.)	0.79 (0.61, 1.02)	0.96 (0.75, 1.22)	0.98 (0.77, 1.24)	0.98 (0.77, 1.25)	0.607
Low HDL-cholesterol	1.00 (ref.)	0.95 (0.77, 1.17)	1.05 (0.86, 1.27)	1.03 (0.85, 1.27)	1.04 (0.85, 1.27)	0.542
Elevated triglycerides	1.00 (ref.)	0.95 (0.80, 1.12)	0.95 (0.80, 1.12)	0.81 (0.69, 0.96)^*^	0.82 (0.69, 0.97)^*^	0.008
**Women**						
Elevated total cholesterol	1.00 (ref.)	0.94 (0.82, 1.08)	0.99 (0.86, 1.14)	1.07 (0.93, 1.23)	0.97 (0.84, 1.12)	0.906
Elevated LDL-cholesterol	1.00 (ref.)	1.16 (0.88, 1.53)	1.01 (0.75, 1.36)	1.21 (0.91, 1.60)	1.02 (0.76, 1.37)	0.975
Low HDL-cholesterol	1.00 (ref.)	0.89 (0.77, 1.04)	0.99 (0.85, 1.16)	0.92 (0.80, 1.06)	1.01 (0.87, 1.17)	0.666
Elevated triglycerides	1.00 (ref.)	0.95 (0.78, 1.15)	0.92 (0.75, 1.12)	0.98 (0.81, 1.19)	0.86 (0.70, 1.04)	0.169

All analyses accounted for the complex sampling design effect and appropriate sampling weights

*Abbreviations*: *LDL* low-density lipoprotein, *HDL* high-density lipoprotein

* *P* < 0.05

** *P* < 0.01

*** *P* < 0.001

^a^ Odds ratios (95% confidence intervals) and *P* for trend were obtained from the multiple logistic regression analysis across quintiles of dietary pattern scores after adjustment for age, residence, household income, education, current smoking, current alcohol consumption, physical activity, total energy intake, body mass index, and menopausal status

^b^ Elevated total cholesterol (≥ 200 mg/dL)

^c^ Elevated LDL-cholesterol (≥ 130 mg/dL)

^d^ Low HDL-cholesterol (men < 40 mg/dL, women < 50 mg/dL)

^e^ Elevated triglycerides (≥ 150 mg/dL)

## Discussion

In this study, three dietary patterns that explain the intake of fatty acids in Korean adults were extracted using the RRR method. Dietary pattern 1 showed high consumption of vegetable oil, legumes, nuts, and fish, dietary pattern 2 was positively associated with consumption of red meat, milk and dairy products, and bread and snacks, and dietary pattern 3 showed higher factor loading for fish. Furthermore, this study examined associations of dietary patterns with lipid disorders. As the score of dietary pattern 2 increased in Korean women, the OR for elevated total cholesterol increased, whereas that for low HDL-cholesterol decreased. In men, dietary patterns 2 and 3 were inversely associated with elevated triglycerides.

Because the RRR method incorporates disease-related nutrients and biochemical indices as response variables and identifies combinations of food intakes explaining maximal variation in these variables, it demonstrates higher correlations between dietary patterns and diseases compared to other dietary pattern analysis methods, such as factor or cluster analysis [31]. Utilizing the RRR method, this study has identified dietary patterns associated with fatty acid intake, known to influence blood lipid profiles, and examined associations with lipid disorders. Consequently, these findings could be instrumental in offering effective, practical dietary guidelines on fatty acid consumption for the prevention and management of lipid disorders.

Dietary pattern 3 which was rich in omega-3 fatty acids and characterized by a higher intakes of fish and milk and dairy products was inversely associated with elevated triglycerides in Korean men. This finding is in accordance with those of previous studies on fish intake and hypertriglyceridemia. In a recent meta-analysis of clinical studies, the triglycerides levels decreased by 0.11 mmol/L in intervention groups that consumed oily fish (*P* = 0.002), whereas no significant changes were observed in control groups [39]. Accordingly, a study that followed 12,981 Norwegian adults aged 30–87 years for 13 years showed that the group of individuals who ate lean fish at least once a week had significantly lower triglycerides levels compared to those who ate lean fish less than once a week [40]. In a 25-year follow-up cohort study of American young adults, the incidence of hypertriglyceridemia was significantly lower in the group of individuals who ate non-fried fish at least once a week than in those who ate it less than once a month [32]. Similar results have been reported in Korean studies. Specifically, in a Korean cohort study, which tracked 3,504 adults aged 40–69 years, men who consumed fish daily exhibited a significantly lower incidence of hypertriglyceridemia compared to those who consumed fish less than once weekly (relative risk [RR] = 0.54, 95% CI = 0.34–0.86, *P* < 0.01), whereas no significant association was observed in women [41]. In another cohort study of 20,670 Korean adults aged 40–69 years, as intake of oily fish increased, the risk of hypertriglyceridemia decreased in both men and women (Q5 vs. Q1: RR = 0.75, 95% CI = 0.60–0.95, *P* = 0.0121 in men; RR = 0.81, 95% CI = 0.69–0.96, *P* = 0.0110 in women) [42]. Fish is an important source of long-chain omega-3 fatty acids, such as EPA and DHA, and is rich in protein. Moreover, because fish contains high amounts of other nutrients, such as vitamins B and D, calcium, iodine, and selenium, it has shown a protective effect on various metabolic diseases, including lipid disorders [43, 44]. The American Heart Association suggests consuming 250 mg of EPA + DHA per day and at least one to two meals with oily fish (3.5 oz per meal) per week to prevent cardiovascular diseases [34]. The clinical guidelines for dyslipidemia presented by the Korean Society of Lipid and Atherosclerosis also recommend consuming fish instead of red or processed meat to improve lipid levels [36].

The inverse association between dietary pattern 3 and elevated triglycerides observed in this study was also accounted for higher intakes of omega-3 fatty acids and milk and dairy products. Omega-3 fatty acids have consistently shown inverse associations with lipid disorders and cardiovascular diseases [9–11, 33, 34]. A higher intake of omega-3 fatty acids has been reported to inhibit the synthesis of triglycerides and very low-density lipoproptein in the liver, which result in preventing hypertriglyceridemia [33]. In addition, intake of omega-3 fatty acids have beneficial effects on lipid profiles through suppressing inflammation as well as the synthesis of thromboxanes and atherosclerotic plaque [34]. Although dairy products have shown different associations with lipid profiles by fat content of dairy or by specific dairy foods, the recent meta-analysis of epidemiologic studies reported that intake of total dairy foods was inversely associated with hypertriglyceridemia in a dose-repsonse manner [45]. In addition, a Women’s Health Initiative study of 35,352 postmenopausal women showed that a higher intake of total dairy foods was associated with a lower concentration of triglycerides [46]. The potential protective effects of dairy foods, such as milk, yogurt, and cheese, on hypertriglyceridemia could be explained through their favourable nutrient content. Calcium combines with fatty acids and decreases fat absorption and thus lowers triglycerides level [47]. Calcium intake from dairy foods also might affect lipid profiles by decreasing intracellular calcium concentration which results in inhibiting fatty acids synthesis and stimulating lipolysis [45]. Amino acids, SFA, and MUFA in dairy products have been suggested to have anti-inflammatory properties, improve insulin sensitivity, and regulate blood lipid profiles [47]. Especially, fermented dairy products contain different probiotic bacteria that may alter gut microbiota and thus exhibit beneficial influences on lipid metabolism [47].

As the score of dietary pattern 2 in this study increased, the risk of having hypercholesterolemia in women increased. Dietary pattern 2 mainly comprised red meat, bread and snacks, and milk and dairy products, and individuals with a higher score of dietary pattern 2 showed high intakes of total fats and SFA. Previous meta-analyses reported that intake of SFA was positively associated with total cholesterol levels [6, 8]. In previous studies using the RRR method, dietary patterns explaining SFA intake were positively associated with incidences of hypercholesterolemia [21] and cardiovascular diseases [22]. Furthermore, a cohort study on the dietary patterns of Korean adults extracted using factor analysis showed that, as the score of the dietary pattern with high intake of flour and meat products increased, the risk of hypercholesterolemia significantly increased in both men and women [48]. A cross-sectional study on Korean women revealed a positive association between Western dietary patterns, characterized by high consumption of meat, bread, and snacks, and the prevalence of hypercholesterolemia, as well as hyper-LDL-cholesterolemia [49]. In a cohort study on northern Chinese adults, the dietary pattern characterized by high amounts of snacks was positively associated with the incidence of hypercholesterolemia [50]. According to a prospective cohort study conducted in Australia, a higher intake of the Western dietary pattern was associated with increased risks of elevated LDL-cholesterol and triglycerides, as well as higher total cholesterol:HDL-cholesterol and triglycerides:HDL-cholesterol ratios [51]. A cross-sectional study involving 4,202 young adults in Southern Brazil found that a dietary pattern primarily comprising processed foods was positively associated with LDL-cholesterol, HDL-cholesterol, and total cholesterol in men [52]. Additionally, a study of Italian adults indicated that a diet rich in soft drinks, fried foods, seed oils, cured meats, butter, red meat, and sweets was linked to a higher triglycerides/HDL-cholesterol ratio [53].

Conversely, this study revealed an inverse association between dietary pattern 2 score and low HDL-cholesterol in women, as well as elevated triglycerides in men. While these associations remain unclarified, they may relate to carbohydrate intake. Notably, as the dietary pattern 2 score increased, the proportion of energy from carbohydrates significantly declined (from 76.0% E in Q1 to 49.3% E in Q5 for men, and from 77.2% E in Q1 to 50.2% E in Q5 for women). Previous studies involving Korean adults have shown an inverse relationship between the Western dietary pattern and hypo-HDL-cholesterolemia risk [49, 54]. In addition, high carbohydrate intake in Korean adults consistently showed positive associations with elevated triglycerides and low HDL-cholesterol [55, 56]. Based on different associations of dietary pattern 2 with each type of lipid disorder, specific dietary strategies should be considered according to individuals’ lipid profiles.

This study’s examination of dietary patterns, notably regarding associations with lipid disorders, was distinctly divided into separate analyses for men and women. This method was guided by previous research [37] illustrating sex-based differences in lipid metabolism. Furthermore, findings from a review article, emphasizing considerable sex-related variations in lipid disorders, atherosclerosis, and cardiovascular disease, also substantiated this approach [38]. This background becomes particularly significant in light of the results for dietary pattern 2, which revealed distinct associations with triglycerides levels in men and women. These findings underscore the imperative for sex-specific dietary considerations in both evaluating and managing lipid disorders, potentially informing future research and clinical practices in this area.

Dietary pattern 1, identified in this study, was characterized by higher factor loadings for vegetable oils and seasonings and showed positive factor loadings for legumes, fish, nuts, and vegetables, paralleling prudent dietary patterns. However, this study found no association between dietary pattern 1 and lipid disorders. Although dietary pattern 1 contained healthy food components, dietary pattern 1 was more closely associated with vegetable oils and seasonings. In addition, the difference in SFA intake for dietary pattern 1 was not significant, with Q1 and Q5 showing 9.4% E vs. 5.9% E in men and 9.5% E vs. 6.0% E in women, while dietary pattern 2 exhibited a more notable difference between Q1 and Q5 (3.2% E vs. 11.0% E in men and 3.0% E vs. 11.2% E in women). Contrasting with these findings, other previous studies have consistently demonstrated the benefits of prudent dietary patterns, rich in fruits, vegetables, legumes, nuts, and fish, against lipid disorders [51, 57]. In previous studies of Korean adults, prudent dietary patterns were inversely associated with dyslipidemia, hypo-HDL-cholesterolemia, and hypertriglyceridemia [48, 58, 59]. Based on this scientific evidence, several dietary guidelines for the management of dyslipidemia recommend dietary patterns rich in whole grains, fruits, vegetables, legumes, and fish [5, 36, 60]. Additional studies should be conducted to investigate dietary patterns beneficial to the prevention and management of lipid disorders in Korean adults.

### Study strengths and limitations

The strength of this study lies in its inclusion of a relatively large sample that is representative of Korean adults. Notably, it is among the first to assess dietary patterns linked to fatty acid intake using the RRR method and to explore the association with lipid disorders. However, certain limitations warrant consideration. As a cross-sectional study using data from the KNHANES, establishing a causal relationship between dietary patterns and lipid disorders is challenging. Potential confounding variables remains and influences from uninvestigated conditions, such as gastrointestinal disorders affecting lipid absorption, could not be considered to examine the impact of dietary patterns on lipid profiles. Moreover, dietary patterns were derived from food intake data collected via a 24-h recall of a single day, rendering it challenging to assert that the data accurately reflect the usual food intake.

## Conclusions

In the current study, dietary patterns explaining the intake of various types of fatty acids were differentially associated with lipid disorders in Korean adults. Dietary pattern 2 which was characterized by higher intakes of red meat, milk and dairy products, and bread and snacks showed a positive association with elevated total cholesterol but an inverse association with low HDL-cholesterol in Korean women. Dietary pattern 3 rich in fish consumption was negatively associated with elevated triglycerides in Korean men. However, dietary pattern 1 which as characterized by higher consumption of vegetable oil, legumes, fish, and nuts but lower intakes of milk and dairy products and red meat showed no association with lipid disorders in Korean adults. These findings could be instrumental in developing dietary guidance and strategies for preventing and managing lipid disorders in this population. Furthermore, they can be used as basic data for providing efficient nutrition education and nutrient intervention to care for patients with lipid disorders. Future studies are needed to demonstrate whether the difference in dietary patterns translates into different clinical outcomes, such as dyslipidemia, type 2 diabetes, or cardiovascular diseases. Prospective cohort or intervention studies are also needed to identify dietary patterns affecting lipid disorders and elucidate their underlying mechanisms.

## Data Availability

The datasets generated and analyzed in this study are available from the Korea National Health and Nutrition Examination Survey (KNHANES) data repository, https://knhanes.kdca.go.kr/knhanes/sub03/sub03_02_05.do (accessed on March 7, 2022).

## Abbreviations

SFA: Saturated fatty acids
PUFA: Polyunsaturated fatty acids
LDL: Low-density lipoprotein
HDL: High-density lipoprotein
EPA: Eicosapentaenoic acid
DHA: Docosahexaenoic acid
RRR: Reduced rank regression
KNHANES: Korea National Health and Nutrition Examination Survey
KDCA: Korea Disease Control and Prevention Agency
IRB: Institutional review board
Q: Quintile
BMI: Body mass index
PLS: Partial least squares
SE: Standard error
OR: Odds ratio
CI: Confidence interval
RR: Relative risk
MUFA: Monounsaturated fatty acids
